# Supplementary material for: Itchy E3 Ubiquitin Ligase-Mediated Ubiquitination of Ferritin Light Chain Contributes to Endothelial Ferroptosis in Atherosclerosis
Source: Int J Mol Sci. 2024 Dec 17;25(24):13524. doi: 10.3390/ijms252413524 (PMC11677933; doi:10.3390/ijms252413524)
Supplement: Supplementary file 1 [file ijms-25-13524-s001.zip › ijms-3367072-supplementary.pdf]

Supplemental Materials and Methods

Materials

Oxidized low-density lipoprotein (ox-LDL, Cat. No. YB-002) was sourced from Yiyuan Biotechnologies, Guangzhou, China. Cycloheximide (CHX, Cat. No. 508739), chloroquine (Cat. No. C6628), and 3-methyladenine (3-MA, Cat. No. M9281) were procured from Sigma-Aldrich. MG132 (Cat. No. ab141003) was sourced from Abcam Inc. (Cambridge, MA, USA).

Animal experiment

Eight-week-old male LDLr$^{-/-}$ mice (Cat. No. T001464, GemPharmatech, Nanjing, China) were randomly assigned to either the control group or the AS model group. The control group mice were provided with a standard chow diet (Cat. No. XTI01WC-009, Xietong Shengwu, Nanjing, China). In the AS model cohort, the mice were administered a high-fat diet enriched with 0.21% cholesterol and 21% fat (D12079B, Open Source Diets, Research Diets, Inc., Changzhou, China). After 12 weeks, mice underwent an overnight fast, followed by blood collection from the orbital cavity. After that, the mice were euthanized by CO_2 inhalation, and the aortic tissues were removed from all groups for further measurement.

In ITCH knockdown studies, LDLr$^{-/-}$ mice were randomly allocated into three groups: control group + lentivirus-scramble-shRNA (control+sh NC), AS model + lentivirus-scramble-shRNA (AS+sh NC) and AS model + lentivirus-ITCH-shRNA (AS+sh ITCH) group. Lentivirus-scramble-shRNA and lentivirus-ITCH-

shRNA (5’-AATCCAGACCACCTGAAATAC-3’) were purchased from GenePharma (Shanghai, China). Mice simultaneously received injection with sh NC or sh ITCH lentiviral particles (2× 10^7TU/mouse) via the tail vein once four weeks. After 12 weeks, the mice were sacrificed as mentioned above.

ELISA

Serum ox-LDL levels were quantified with ELISA kits following the manufacturer's instructions (Cat. No. F11344, Shanghai Westang Biotechnology Co. Ltd, Shanghai, China). The levels of inflammatory cytokines TNF-α (Cat. No. PT518), IL-6 (Cat. No. PI330), and IL-1β (Cat. No. PI305) were assessed using ELISA kits from Beyotime Biotechnology Co. Ltd (Nanjing, China), according to the manufacturer's instructions.

Quantification of atherosclerosis and serum lipid profile

Plaque severity was assessed using serial sections from the aortic root, following the method established in our previous study [24]. Serum levels of total cholesterol (TC, Cat. No. A111-1-1), triglycerides (TG, Cat. No. A110-1-1), low-density lipoprotein cholesterol (LDL-c, Cat. No. A113-1-1), and high-density lipoprotein cholesterol (HDL-c, Cat. No. A112-1-1) were measured using a biochemical assay kit (Nanjing Jiancheng Bioengineering insititue, Nanjing, China), as described in our earlier publications [24].

Immunofluorescence staining of aorta

Cryosections of the thoracic aorta were employed for immunofluorescence staining using appropriate antibodies such as anti-FTL (68068-1-Ig, ProteinTech, dilution at 1:800) and anti-CD31 (CL488-80530, ProteinTech, dilution at 1:500). Fluorescence intensity was observed using a fluorescence microscope (Olympus, Tokyo, Japan) and quantified with Image-Pro Plus analysis software version 6.0.

Cell culture

Human aortic endothelial cells (HAECs) sourced from ScienCell Research Laboratories (Cat. No. #6100) were cultured in endothelial cell medium as described in our previously established method [25]. Cells within passages three to eight were utilized for these experiments.

Transfection

To knockdown ITCH, HAECs were transfected with 100 nM of ITCH siRNA or control siRNA (GenePharma RNAi, Shanghai, China) using Lipofectamine 3000, following the manufacturer's instructions, for a duration of 24 hours. Forward primer sequence for ITCH siRNA is 5'-GCUGUUGUUUGCCAUAGAATT-3', and reverse primer sequence is 5'-UUCUAUGGCAAACAACAGCTT-3'. To detect the role of ITCH on ox-LDL-induced endothelial injury and ferroptosis, HAECs were transfected with either

100 nM control siRNA (con siRNA) or 100 nM ITCH siRNA for 24 hours, and subsequently treated with 100 μg/mL ox-LDL for another 24 hours. To investigate the role of FTL in the impact of ITCH on ox-LDL-induced endothelial injury and ferroptosis, cells were first transfected with ITCH siRNA, then subsequently infected with 100 nM FTL siRNA for 24 hours, and finally exposed to ox-LDL for an additional 24 hours. Forward primer sequence for FTL is 5'-CCUGGAGACUCACUUCCUATT-3', and reverse primer sequence is 5'-UAGGAAGUGAGUCUCCAGGAA-3'.

Cell viability assay

The HAECs ($1×10^4$ cells /mL) were cultured in a 96-well plate, and cell viability was determined with an MTT assay (Cat. No.C0009, Beyotime, Shanghai, China) following the protocol outlined in an earlier study [26].

Lactate dehydrogenase (LDH) assay

Cell injury was determined by assessing LDH release via a colorimetric LDH cytotoxicity detection kit, in accordance with the manufacturer's guidelines (Cat. No.C0016, Beyotime, Shanghai, China).

Nitric Oxide (NO) assay

NO production was assessed using the Griess reaction with an NO detection kit (Cat. No. S0021, Beyotime Biotech Inc., Nanjing, China) as

previously described [27].

Monocyte Adhesion Assay

Cell adhesion experiments were conducted following previously established methods [28]. Before the end of treatment, cell treatment was discontinued, followed by the addition of calcein-labeled THP-1 monocytes to facilitate binding for one hour. Subsequently, fluorescence intensity was quantified using a microplate reader (Synergy H1, BioTek Instruments Inc., Winooski, VT, USA) at an excitation wavelength of 485 nm and an emission wavelength of 520 nm.

Ferrous iron measurement

Ferrous iron contents were measured using an iron assay (ab83366, Abcam) and were performed according to the manufacturers' instruction as previously described [29]. In brief, the supplied probe bound to ferrous ions released by the cells, and the resulting absorbance at 593 nm was measured to quantify the intracellular ferrous content.

Glutathione (GSH) assay

GSH levels were assessed using a GSH assay kit (A006-2, Jiancheng, Nanjing, China) according to the manufacturer's guidelines as described previously [30]. The interaction between 5,5'-dithiobis(2-nitrobenzoic acid) and GSH results in the formation of a yellow chromogen, with its absorbance being

recorded at 410 nm.

Lipid peroxidation quantification by C11-BODIPY581/591, lipid peroxidase (LPO), malondialdehyde (MDA) assay

Lipid peroxidation was assessed using the lipid reactive oxygen species (ROS) fluorescent probe C11-BODIPY581/591 (Invitrogen™, Carlsbad, CA, USA) [26]. A stock solution of the probe was added to the culture medium to achieve a concentration of 10 μM. When oxidized by free radicals, the fluorescence emission of the probe changes from red to green, observable under fluorescence microscopy. The ratio of green fluorescence intensity (510 nm) to red fluorescence intensity (590 nm) was used to quantify lipid droplet fluorescence, measured with a microplate reader (Synergy H1, BioTek, Winooski, VT, USA). And the data are expressed as a percentage relative to the control.

Lipid peroxidase (LPO) was evaluated by determining the intracellular concentration using a commercial kit, adhering to the provided protocol (#A106, Nanjing Jiancheng Bioengineering Institute, Nanjing, China).

To measure MDA , a thiobarbituric acid assay kit (Cat. No. S0131) from Beyotime (Shanghai, China) was used.

Quantitative real-time-PCR

Quantitative real-time PCR (qRT-PCR) analysis was conducted following

methods established in our previous studies [24, 25]. The sequences of the primers utilized were shown in Supplementary Table 1.

Western blot and immunoprecipitation *analysis*

Western blot and immunoprecipitation analyses were performed following our previously established methods [25]. The Whole cell extracts from tissue and cultured cells were lysed using NP-40 Lysis Buffer kit (Cat. No. P0013F, Beyotime biotechnology Co. Ltd, Shanghai, China). For western blot, equivalent quantities of protein extracts underwent separation on sodium dodecyl sulfate polyacrylamide gels and then transferred to nitrocellulose membranes. Subsequently, the membranes were incubated with the appropriate primary antibodies at 4 °C for an extended period (Detailed in Supplementary Table 2). After this step, the membranes were exposed to IRDye 680RD secondary antibodies. For immunoprecipitation, cellular extracts were incubated with the described antibodies for overnight, followed by 2-hour incubation with protein G plus-agarose (Cat. No. P2053, Beyotime biotechnology Co. Ltd, Shanghai, China). The immunoprecipitated products underwent washing with immunoprecipitation buffer prior to being resolved by SDS-PAGE. The designated antibodies were used to visualize the proteins.

2.17. Statistical analysis

The data are expressed as mean ± standard deviation. Statistical analysis

employed GraphPad Prism 8 software. The Shapiro-Wilk test was used to assess the normality of the data. After confirming nor-mal distribution, an unpaired Student's t-test was used to compare two groups and one-way ANOVA with the Newman–Keuls test for multiple groups, with statistical significance defined as $P < 0.05$.

Supplementary Table S1. Primer sequences of genes

**Gene**		**Primer sequence**
FTL (mouse)	Forward	5′- CCACCGAGGTGGAAGC-3′
	Reverse	5′- CCTCCTTATCCAGATAG-3′
FTL (human)	Forward	5′-CAGCCTGGTCAATTTGTACCT -3′
	Reverse	5′- GCCAATTCGCGGAAGAAGTG -3′
TNF-α (human)	Forward	5′- GTCACTCATTGCTGAGCCTCT -3′
	Reverse	5′- AGCTTCTTCCCACCCACAAG -3′
IL-6 (human)	Forward	5′-GCCGCCCCACACAGACA-3′
	Reverse	5′-CCGTCGAGGATGTACCGAAT-3′
IL-1β (human)	Forward	5′-CACGATGCACCTGTACGATCA-3′
	Reverse	5′-GTTGCTCCATATCCTGTCCCT-3′
TNF-α (mouse)	Forward	5′-CGTCAGCCGATTTGCTATCT-3′
	Reverse	5′-CGGACTCCGCAAAGTCTAAG-3′
IL-6 (mouse)	Forward	5′-TGGAGTCACAGAAGGAGTGGCTAAG-3′
	Reverse	5′-TCTGACCACAGTGAGGAATGTCCAC-3′
IL-1β (mouse)	Forward	5′-ATGAGAGCATCCAGCTTCAA-3′

	Reverse	5′-TGAAGGAAAAGAAGGTGCTC-3′
β-actin (human)	Forward	5′- CATGTACGTTGCTATCCAGGC -3′
	Reverse	5′- CTCCTTAATGTCACGCACGAT -3′
β-actin (mouse)	Reverse	5′- GGCTGTATTCCCCTCCATCG -3′
	Forward	5′- CCAGTTGGTAACAATGCCATGT-3′

Supplemental Table S2 Antibodies for Western blotting

Antibody	Company	Country	Cat. No.	Dilution
Ub	Cell singaling	USA	#3936	1:1000
SLC7A11	ABCAM	USA	ab175186	1:1000
FTH	ABCAM	USA	ab65080	1:1000
VCAM-1	ProteinTech	China	30958-1-AP	1:2000
GPX4	ProteinTech	China	67763-1-Ig	1:1000
FTL	ProteinTech	China	10727-1-AP	1:2000
ITCH	ProteinTech	China	20920-1-AP	1:1000
β -actin	ProteinTech	China	66009-1-Ig	1:20000
GAPDH	Kangchen	China	kc5G4	1:10000
